# Influence of Processing Parameters on Additively Manufactured Architected Cellular Metals: Emphasis on Biomedical Applications

**DOI:** 10.3390/jfb16020053

**Published:** 2025-02-08

**Authors:** Yixuan Shi, Yuzhe Zheng, Chengcong Huang, Shangyan Zhao, Xuan Li, Yuchen Lu, Yuzhi Wu, Peipei Li, Luning Wang, Yageng Li

**Affiliations:** 1 Beijing Advanced Innovation Center for Materials Genome Engineering, School of Materials Science and Engineering, University of Science and Technology Beijing, Beijing 100083, China; d202210281@xs.ustb.edu.cn (Y.S.); qzkhzyz2022@163.com (Y.Z.); d202310310@xs.ustb.edu.cn (C.H.); d202410300@xs.ustb.edu.cn (S.Z.); d202410299@xs.ustb.edu.cn (X.L.); luyuchen0420@foxmail.com (Y.L.); m202310373@xs.ustb.edu.cn (Y.W.); luning.wang@ustb.edu.cn (L.W.); 2 Institute of Materials Intelligent Technology, Liaoning Academy of Materials, Shenyang 110004, China; 3 School of Mechanical Engineering, University of Science and Technology Beijing, Beijing 100083, China; lipeipei@ustb.edu.cn; 4 Beijing Key Laboratory of Lightweight Metal Forming, Beijing 100083, China

**Keywords:** additive manufacturing, cellular structure, processing parameter, biomaterial

## Abstract

Laser powder bed fusion (LPBF) has emerged as a transformative additive manufacturing technique for fabricating architected cellular metallic structures, offering tailored properties for diverse biomedical applications. These structures are particularly well-suited for bone implants, scaffolds, and other load-bearing medical devices due to their ability to achieve lightweight designs, enhanced mechanical properties, and customized geometries. However, the complex interactions between LPBF process parameters and the resulting structural and mechanical properties pose significant challenges in achieving the precision and reliability required for clinical applications. This review provides a comprehensive analysis of the effects of LPBF process parameters, including laser power, scanning speed, and layer thickness, on key attributes such as dimensional accuracy, density, surface roughness, and microstructure. Their influence on the mechanical performance, including strength, fatigue resistance, and functional properties, is critically examined, with specific attention to biomedical relevance. The impact of lattice design factors, such as topology, unit cell size, and orientation, is also discussed, underscoring their role in optimizing biocompatibility and structural integrity for medical applications. Challenges such as surface defects, geometric inaccuracies, and microstructural inconsistencies are highlighted as key barriers to the broader adoption of LPBF in biomedical fields. Future perspectives focus on advancing LPBF technologies through process optimization and integration with advanced computational tools, such as machine learning, to enable efficient manufacturing of complex, patient-specific architectures. By addressing these challenges, LPBF has the potential to revolutionize the development of next-generation biomaterials, tailored to meet evolving clinical needs and improve patient outcomes.

## 1. Introduction

Metallic cellular structures have emerged as a transformative class of materials in the biomedical field, owing to their unique combination of large surface area, low density, and tailored mechanical properties [1]. These structures are designed with intricate, repeating unit cells that mimic the architecture of natural bone, enabling optimal load distribution and minimizing stress shielding effects when used as implants [2]. Furthermore, their large surface area and interconnected porosity facilitate bone ingrowth and vascularization, promoting long-term integration with native tissues [3]. Beyond orthopedic applications, metallic cellular structures are gaining traction in cardiovascular and dental implants, due to their ability to be customized for patient-specific geometries and functional requirements [4,5]. Advances in additive manufacturing (AM) have revolutionized the fabrication of these architectures, allowing for unprecedented control over microstructural features and enabling the realization of complex designs that were previously unattainable [6,7,8,9,10]. This synergy between cellular structure design and AM technology holds immense potential for addressing critical challenges in biocompatibility, mechanical compatibility, and biodegradability.

**Figure 1:**
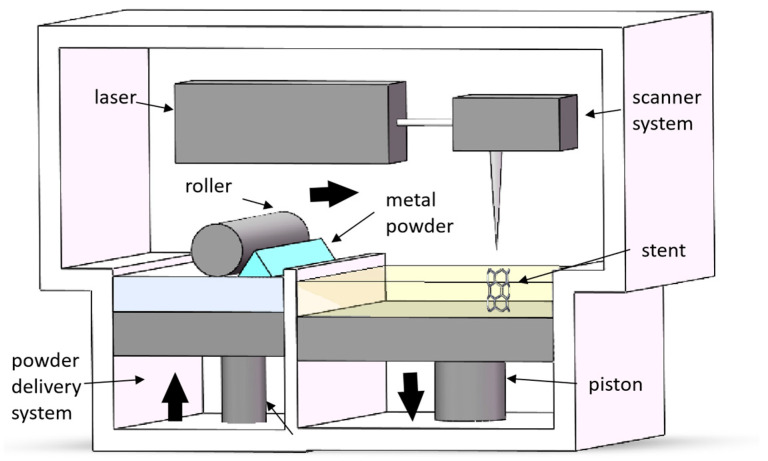
Schematic diagram of the LPBF process. (Adapted with permission from Ref. [4]. Copyright 2023 Elsevier).

Among the various AM techniques, laser powder bed fusion (LPBF) has emerged as a leading method for fabricating metallic architected structures, due to its precision and design flexibility [11]. LPBF utilizes a high-energy laser to selectively melt metallic powder layer by layer, enabling the creation of complex geometries with exceptional accuracy and repeatability (Figure 1) [4,12]. This layer-wise approach is particularly suited for producing architected cellular structures with intricate features and controlled porosity. Compared to conventional manufacturing techniques, LPBF allows for the direct fabrication of near-net-shape components, reducing material waste and post-processing requirements [13]. Furthermore, LPBF offers unparalleled control over microstructural features through precise tuning of processing parameters [14]. This capability enables the optimization of mechanical properties, including strength, stiffness, and fatigue resistance, which are critical for biomedical applications [15]. The compatibility of LPBF with a wide range of biocompatible metals, coupled with its ability to fabricate patient-specific designs, makes it a promising tool for advancing personalized medicine [16,17,18,19,20,21].

The quality and performance of LPBF architected cellular metals are profoundly influenced by the selection and optimization of processing parameters. Parameters such as laser power, scanning speed, hatch spacing, layer thickness, and build orientation directly impact the printing accuracy, surface roughness, and microstructure of the fabricated components [22]. Precise control of these parameters is critical for achieving the desired geometrical fidelity of architected cellular structures, which are often characterized by intricate and repeating unit cells. Deviations in processing conditions can lead to dimensional inaccuracies, incomplete fusion, or residual stresses, compromising the structural integrity of the final product [23]. Surface roughness, which is another crucial factor, affects not only the mechanical behavior but also the biological performance [24]. At the microstructural level, processing parameters govern the solidification behavior, grain morphology, and phase distribution, which collectively determine the mechanical properties, such as strength, stiffness, and ductility. For biomedical applications, where safety and functionality are paramount, the optimization of LPBF parameters ensures the production of implants with consistent quality, tailored mechanical behavior, and enhanced biocompatibility.

Most existing reviews on LPBF primarily emphasize the influences of processing parameters on the properties of bulk metals [25,26,27]. While these studies provide valuable insights, bulk metals and architected cellular metals exhibit fundamentally different characteristics due to their distinct structural configurations. Bulk metals are solid and continuous, with their properties predominantly governed by material composition and microstructure. In contrast, architected cellular metals incorporate designed porosity and complex unit cell geometries, resulting in unique mechanical behaviors, such as tunable stiffness, enhanced energy absorption, and anisotropic properties. The interplay between processing parameters and these geometrical features introduces additional complexities in understanding and optimizing their performance. Surface roughness, dimensional accuracy, and microstructural heterogeneity play even more critical roles in cellular structures, as they directly affect mechanical behavior, fatigue resistance, and biological responses in biomedical applications. Despite the growing interest in architected cellular metals, there remains a gap in comprehensive reviews dedicated to the effects of LPBF processing parameters on their properties. In this review, we aim to bridge this gap by systematically exploring how processing parameters influence the quality, mechanical properties, and performance of LPBF-fabricated metallic architected cellular structures, with a particular focus on their biomedical applications.

## 2. Process Parameters in the LPBF Process

**Figure 2:**
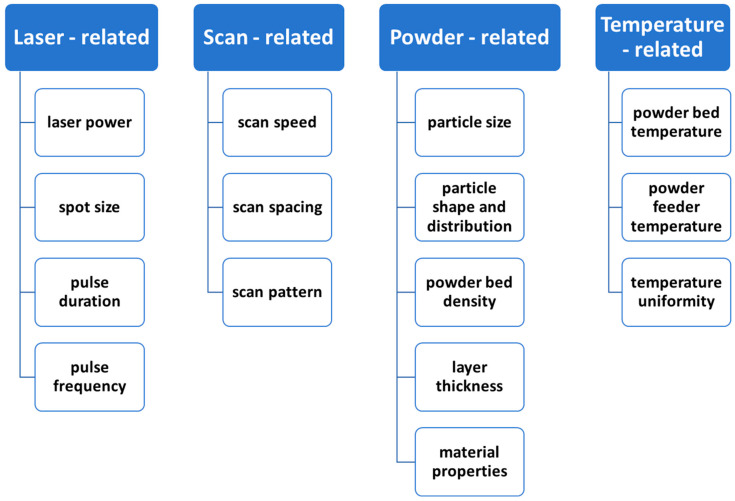
Main process parameters in the LPBF process.

In any manufacturing technology, including LPBF, the selection of process parameters must align closely with the materials to ensure optimal outcomes. The exploration of optimal process parameters to enhance LPBF forming quality has been a persistent focus and challenge in LPBF research. Due to the rapid cooling and solidification inherent in the LPBF process, the interaction between the laser and powder introduces numerous uncertainties. Compared to traditional manufacturing methods, LPBF involves a greater number of variable process parameters, and these parameters interact in complex ways during processing, making optimization particularly intricate. Yadroitsev et al. [28] highlighted the fact that over 130 process parameters in the LPBF process can influence the quality and performance of the final fabricated specimens. These parameters can be broadly categorized into the following four key groups: laser-related, scanning-related, powder-related, and temperature-related factors (Figure 2). Variations in these parameters may lead to different kinds of potential defects and performance issues during the LPBF process. Understanding and optimizing these parameters is critical for advancing the capabilities and reliability of LPBF technology.

### 2.1. Laser-Related Parameters

The performance and reliability of the LPBF system largely depend on the laser-related parameters. Variations in these parameters are key determinants of either high-quality parts or significant defects during the LPBF process. Nagarajan et al. [29] categorized laser-related parameters into several critical factors, including laser type (continuous wave (CW) or pulse wave (PW)), laser power, energy distribution, and spot size.

**Figure 3:**
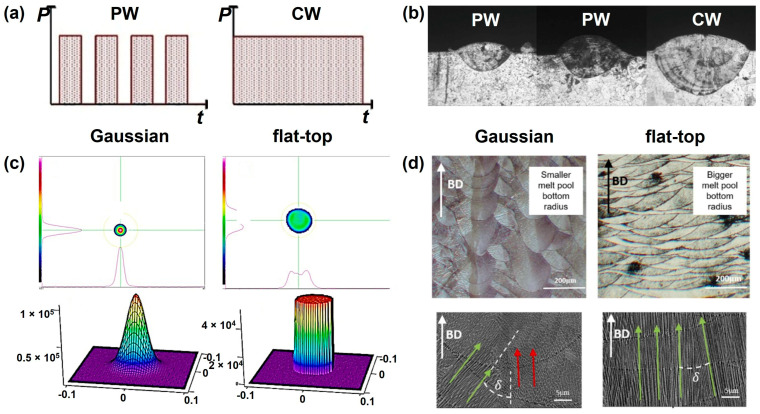
Laser-related parameters: (**a**) pulsed wave (PW) and continuous wave (CW) laser types [30] (*P*: power, *t*: time); (**b**) single track deposition melt pool with different laser types (Adapted with permission from Ref. [30]. Copyright 2019 Elsevier); (**c**) laser beam profile with Gaussian beam and flat-top beams (Adapted with permission from Ref. [35]); (**d**) melt pool characteristics and dendritic growth pattern with different laser beam profiles (δ: crystalline angle; Arrows: crystalline orientation; Colors: heat concentration; Adapted with permission from Ref. [37]).

Research on the impact of laser type is relatively limited. Caprio et al. [30] conducted experimental and modeling studies on the production efficiency of AISI 316 powder processed by LPBF using different laser types (Figure 3a). Their findings revealed that continuous wave lasers significantly enhance the melting capacity (Figure 3b), achieving 2–3 times higher efficiency compared to pulse wave lasers, while also broadening the process window. Expanding on this, Ozsoy et al. [31] investigated energy transfer under LPBF’s pulse mode. They discovered that the laser pulse frequency influences not only component density but also mechanical properties. Specifically, an increased distance between pulse input points, under constant laser energy density and scanning distance, causes localized energy concentration, leading to molten pool collapse and the formation of small pores, ultimately compromising printing quality.

Laser power, one of the most influential parameters in the LPBF process, has been extensively studied. Laakso et al. [32] explored its effect on the density of H13 tool steel components. Their results showed that higher laser power increases the volume energy density (VED), leading to enhanced component strength. Increased laser power widens the molten pool and increases the frequency of remelting/recooling cycles, thereby improving part density. Similarly, Elsayed et al. [33] demonstrated that higher laser power reduces surface roughness and porosity, while improving the elastic modulus and ultimate compressive strength. However, excessive laser power can also induce stress concentration, which reduces the material’s plasticity and elongation.

Laser energy distribution plays a critical role in determining the uniformity of melting and the overall quality of the printed parts [34]. A uniform distribution ensures consistent energy delivery across the powder bed, resulting in homogeneous material properties and a smooth surface finish [35]. The shape and size of the laser beam significantly influence this distribution (Figure 3c). For instance, Liu et al. [36] investigated the effects of laser beam mode on LPBF AlSi10Mg alloy and found that surface quality and relative density of the typical multi-mode LPBF sample were higher than the Gaussian-mode ones. During the LPBF process, molten pools generated by the multi-mode laser beam are controlled by the conduction mode, whereas those generated by the Gaussian laser beam are controlled by the keyhole mode. However, the multi-mode sample may have a strong crystallographic texture. Pérez-Ruiz et al. [37] also found that the beam shape mode provide extra control over the microstructure and mechanical properties of the components manufactured using LPBF. The flat-top laser mode can promote the dendritic growth epitaxially along the layers, facilitating a higher texture index (Figure 3d).

Spot size, another crucial parameter, affects the distribution of laser energy on the powder bed. Maria et al. [38] studied the influence of laser spot diameter on the forming quality of Hastelloy X parts. They observed that high-power lasers with larger beam diameters increased the production efficiency from 6 mm^3^/s to 16 mm^3^/s, a 2.6-fold improvement. Larger spot diameters enhance laser input per unit area, leading to better melting and significantly faster production rates.

In summary, laser-related parameters such as type, power, energy distribution, and spot size critically influence the melting behavior, component density, mechanical properties, and production efficiency of the LPBF process. A thorough understanding and optimization of these parameters is essential to achieving high-quality, defect-free parts that are tailored to specific applications.

### 2.2. Scan-Related Parameters

Scan-related parameters play a pivotal role in determining the quality and performance of parts fabricated using LPBF. These parameters typically include scanning speed, scan spacing, and scan strategy. Adjusting these parameters can significantly impact the melting behavior, microstructure, and mechanical properties of the printed parts, even when other parameters remain fixed.

Scanning speed influences the duration of laser energy interaction with the powder bed. Pal et al. [39] investigated the effects of scanning speed on the densification and metallurgical properties of Ti6Al4V alloy in the LPBF process. Their findings revealed that, as scanning speed increases, the laser energy exposure time per unit area decreases. This insufficient melting–cooling–remelting cycle can lead to a variety of defects, including porosity, surface irregularities, and deformation, such as bending or collapse. Optimizing scanning speed is therefore essential for achieving adequate melting and minimizing defects.

Scan spacing, which refers to the distance between adjacent laser tracks, is another critical parameter. Pupo et al. [40] studied its influence on single-track molten pools and found that smaller scan spacing facilitates the formation of continuous and uniform molten material layers. This improves the overall quality and consistency of LPBF components. However, excessively small scan spacing can increase processing time and may lead to overheating, which could cause distortion or undesirable microstructural changes.

**Figure 4:**
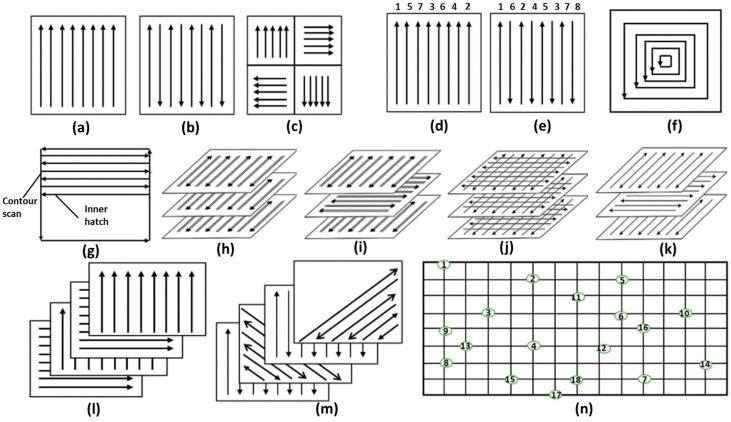
Schematic diagram of different types of scanning strategies [41]: (**a**) unidirectional scan, (**b**) bi-directional/zigzag scan; (**c**) island scan; (**d**) variation of scanning sequences based on unidirectional scan; (**e**) variation of scanning sequences based on bi-directional scan; (**f**) helix scan; (**g**) contour scan; (**h**) bi-directional, double pass of laser beam; (**i**) bi-directional, double pass of laser beam, 90° rotation scan vector between layers; (**j**) cross scan; (**k**) bidirectional, single pass of laser beam, 90° rotation of scan vector between layers; (**l**) 90° rotation of unidirectional scan between successive layers; (**m**) 45° rotation of scan vector; (**n**) point melting scan. (Adapted with permission from Ref. [41]. Copyright 2021 Springer Nature) (Arrows: different direction; Numbers: Scan in sequence).

Among the scan-related parameters, scan strategy has garnered significant research attention due to its profound influence on part density, residual stress, and mechanical properties. Common scanning strategies include unidirectional, bidirectional, island, spiral, and fractal approaches (Figure 4) [41]. Amirjan et al. [42] demonstrated that adopting an island strategy with interlayer rotation or a continuous bidirectional scanning strategy can result in near-full density parts. The rotation between layers promotes a uniform structure and facilitates even temperature distribution, as remelting occurs in different directions during subsequent layer deposition.

Residual stress is another key factor that is influenced by scan strategy. Studies have shown that shorter scan vector lengths are effective in reducing residual stress, which in turn enhances the mechanical properties of LPBF parts [43,44,45]. Strategies such as island scanning, spiral scanning, and fractal scanning typically involve shorter vector lengths and are therefore widely employed in LPBF processing [46,47]. These strategies help to minimize thermal gradients and residual stresses by enabling more uniform heat distribution across the layers.

In summary, scan-related parameters are crucial for controlling the microstructure, mechanical performance, and defect levels in LPBF components. While moderate scanning speed and scan spacing are beneficial, optimizing the scan strategy—especially with approaches such as island or spiral scanning—can further improve part density, residual stress, and overall mechanical properties. A systematic approach to parameter optimization is essential for achieving reliable and high-performance LPBF parts that are tailored to specific applications.

### 2.3. Temperature-Related Parameters

LPBF is a high-temperature manufacturing process in which temperature-related parameters significantly influence the forming quality, stress distribution, and mechanical properties of the fabricated parts. These parameters include the preheating temperature of the powder bed and substrate, as well as the thermal gradients and temperature fields that develop during the printing process.

Preheating the powder bed serves to enhance the fluidity of the powder, facilitating improved powder spreading and melting behavior [48]. The substrate’s preheating temperature directly impacts the thermal gradient and temperature field, which in turn affect the stability of the melt pool and the residual stress within the printed part. Maintaining a constant temperature during the process is critical for achieving uniformity in layer deposition and reducing thermal distortions [49].

**Figure 5:**
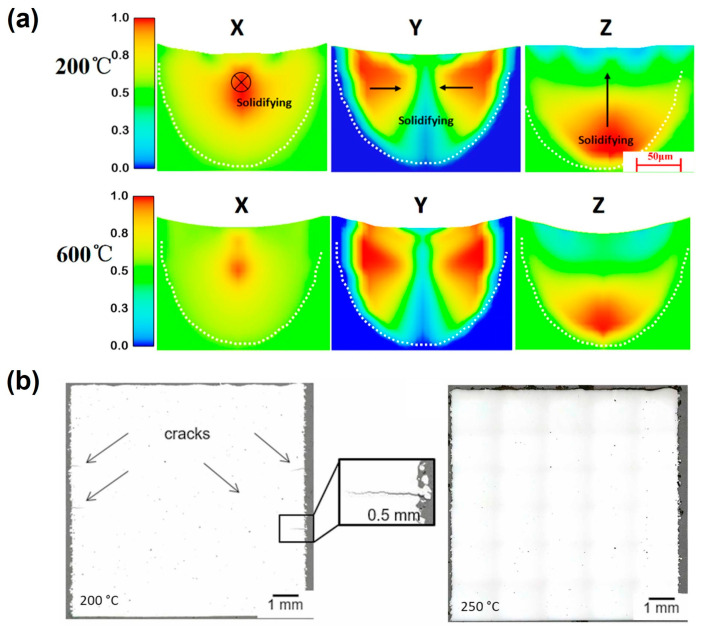
Temperature-related parameters: (**a**) vectorial temperature gradient of LPBF Inconel 738 with different preheating temperatures (Adapted with permission from Ref. [50]); (**b**) defect analysis micrograph of LPBF H10 tool steel with different preheating temperatures (Adapted with permission from Ref. [52]).

Wang et al. [50] investigated the effect of preheating on Inconel 738 using transient numerical simulations and experimental validation. Their results revealed that increasing the preheating temperature significantly reduced the temperature gradient, leading to a deeper and longer melt pool with a higher aspect ratio (Figure 5a). This enhanced the melting process, resulting in improved part quality. Similarly, Savalani et al. [51] studied LPBF of magnesium and demonstrated that preheating the substrate at 180 °C for 30 min greatly stabilized the melt pool, enhancing the overall forming quality of magnesium parts.

Preheating also plays a vital role in mitigating defects such as cracks and porosity. Wild et al. [52] and Waqar et al. [53] explored the effects of various preheating temperatures on different alloys, observing that higher preheating temperatures effectively reduced cracking and other defects (Figure 5b). This improvement is attributed to the decreased thermal gradient, which minimizes thermal stresses and promotes better bonding between layers. Additionally, the temperature control during the LPBF process impacts the grain size, grain morphology, as well as the grain orientations of the materials. Gu et al. [54] found that the complex directions of the large temperature gradients contributed to formation of the unique microstructural transition between columnar and cellular dendrites in the center of the molten pool. While substrate and powder bed preheating are well-researched, further studies are needed to explore advanced temperature management strategies. Techniques such as localized heating, dynamic thermal control, and real-time monitoring could enhance process stability and enable the fabrication of components with tailored properties.

In summary, temperature-related parameters are crucial for achieving high-quality, defect-free LPBF parts. Optimizing these parameters not only improves the forming quality and mechanical properties but also mitigates thermal stresses and defects, paving the way for more reliable and advanced applications of LPBF technology.

### 2.4. Powder-Related Parameters

Powder-related parameters are critical factors that significantly influence the quality of parts produced through LPBF. These parameters can be categorized into powder properties—such as particle size, shape, type, and chemical composition—and operational parameters, including powder bed density and layer thickness. Achieving optimal powder characteristics is essential for ensuring uniform powder spreading, improving flowability, and minimizing the risk of defects during the LPBF process [55].

**Figure 6:**
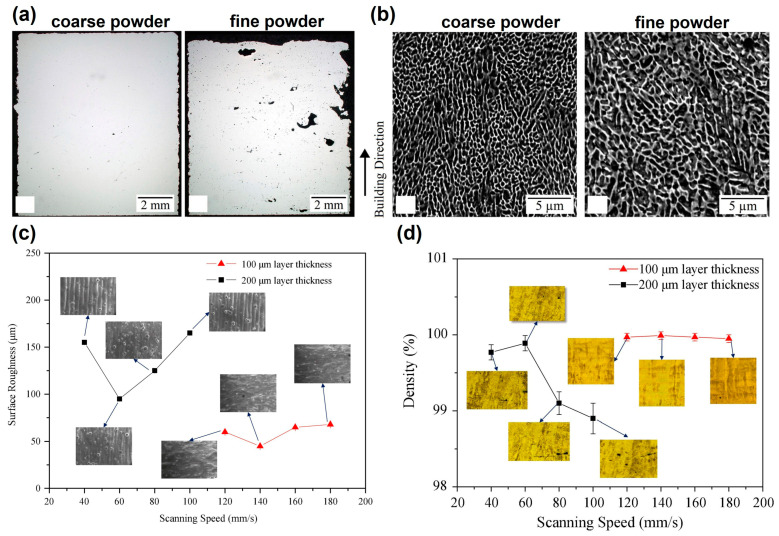
Powder-related parameters: (**a**) cross-sectional optical micrographs of LPBF AlSi10Mg with coarse and fine powders (Adapted with permission from Ref. [57]. Copyright 2021 Elsevier); (**b**) microstructure of LPBF AlSi10Mg with coarse and fine powders [57]; (**c**) surface roughness of LPBF Ti6Al4V fabricated at different layer thicknesses; and (**d**) density of LPBF Ti6Al4V fabricated at different layer thicknesses (Adapted with permission from Ref. [60]. Copyright 2020 Elsevier).

The particle size and its distribution play a pivotal role in powder flowability and laser absorption. Uniformly sized powders with good flowability facilitate smooth layer deposition, ensuring consistent energy absorption and melting during the process. Zhang et al. [56] found that the tungsten powder layer absorptivity diminished with increasing particle size during LPBF. Moreover, Balbaa et al. [57] investigated the role of powder particle size on laser powder bed fusion processability of an AlSi10Mg alloy. The flowability and packing density of the fine powder was lower than that of the coarse powder, leading to lower relative density of the as-build specimens (Figure 6a). Meanwhile, the coarse powder resulted in a finer cellular structure and, consequently, higher microhardness than the fine powder feedstock (Figure 6b).

Powder morphology is another critical aspect. Attar et al. [58] emphasized the importance of initial powder processing and shape, showing that spherical powder particles enhance final part density and mechanical properties compared to irregularly shaped powders. For aluminum-based alloys, Olakanmi et al. [59] highlighted that surface oxidation and irregular particle shapes exacerbate powder agglomeration, increasing the likelihood of pore defects in the fabricated components.

**Figure 7:**
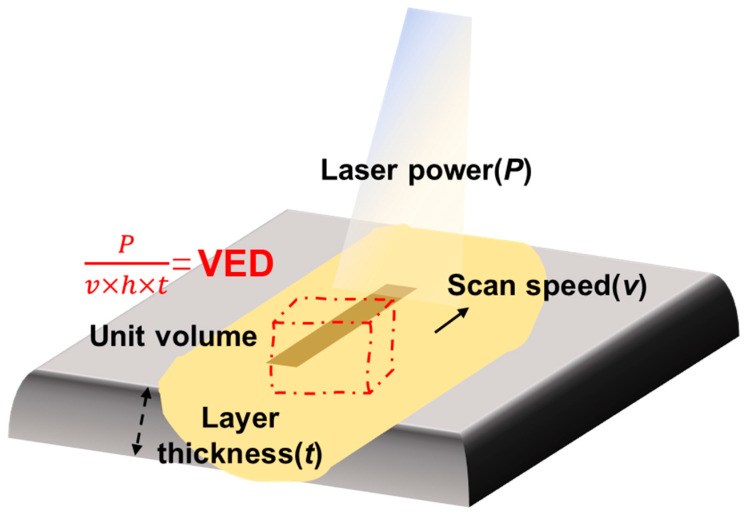
Interactions between laser power, scanning speed, scan spacing, and layer thickness.

Powder bed density and layer thickness are equally influential in determining part quality and production efficiency. Adriano et al. [61] found that increasing the layer thickness by approximately 1.6 times could reduce the manufacturing time by 40% without significantly affecting surface roughness. However, excessive increases in layer thickness can destabilize the scanning trajectory, leading to higher surface roughness and defects, as observed by Shi et al. (Figure 6c,d) [60]. Their research indicated that, while moderate changes in layer thickness have a minimal impact on density and tensile properties, excessive layer thickness can compromise part integrity and dimensional accuracy. Figure 7 illustrates the relationship between layer thickness and laser power, scanning speed, and scanning spacing during the LPBF process. The VED plays a significant role in determining the quality of the LPBF parts.

In addition to these parameters, chemical composition and contamination must be carefully controlled. For example, oxidation on the surface of aluminum alloy powders can degrade part quality by introducing defects and reducing mechanical properties [62]. Ensuring a clean and inert environment during powder storage and handling is essential for preserving powder quality.

In summary, optimizing powder-related parameters—encompassing powder properties and operational conditions—is crucial for achieving high-quality LPBF parts. Future research should focus on advanced powder preparation techniques and real-time monitoring of powder bed properties.

Finally, based on the LPBF process and the associated parameters discussed above, we have compiled a summary of key factors influencing LPBF quality in Table 1, aiming to elucidate the role of each parameter.

## 3. LPBF Architected Cellular Metals

### 3.1. Architected Cellular Structures

Architected cellular structures are characterized by a spatial arrangement of cells with edges and faces, forming two-dimensional or three-dimensional cellular solids [63]. These structures offer significant advantages over traditional solid designs due to their lower mass and superior performance, making them highly versatile across various engineering and biomedical applications. The geometry, materials, and processing technologies used to create these lattice structures have a profound impact on their properties. Traditional manufacturing techniques, such as powder metallurgy, are constrained by limited design freedom and challenges in controlling porosity. However, LPBF overcomes these limitations by enabling intricate structural freedom, making it ideal for fabricating architected cellular structures.

One of the key benefits of architected cellular structures is their programmable mechanical properties, which are closely linked to unit cells and porosity [64]. These properties make lattice structures particularly attractive for biomedical applications, where precise control over mechanical performance and porosity is critical. For example, in bone scaffolds, lattice designs can mimic the mechanical behavior of natural bone while facilitating cellular ingrowth and vascularization [65].

Architected cellular materials typically consist of interconnected beam elements or support rods joined at nodes, with variations in uniformity depending on the lattice boundary and volume [66]. These structures can be broadly categorized into random and periodic topologies. Random architected cellular structures feature cells arranged in a non-systematic probability distribution, resulting in a more stochastic arrangement. In contrast, periodic architected cellular structures have cells systematically organized along independent axes, providing predictable and repeatable mechanical and functional properties.

**Figure 8:**
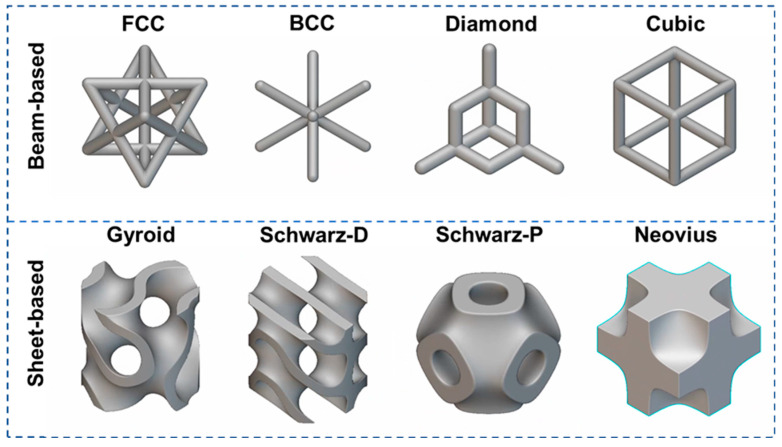
Different types of architected cellular structures.

Currently, commonly studied types of lattice structures include beam-based (e.g., cubic, diamond, BCC, and FCC) and sheet-based (e.g., gyroid, Schwartz P (primitive), and Schwartz D (diamond)) (Figure 8). Each lattice type offers distinct advantages for specific biomedical applications. For instance, triply periodic minimal surface (TPMS) structures are widely favored in bone tissue engineering due to their high surface area and isotropic mechanical properties, which facilitate osteointegration and uniform load distribution [67]. Among TPMS structures, the gyroid stands out as a well-known design, renowned for its high specific strength and stiffness. Furthermore, studies have demonstrated that the gyroid structure exhibits superior osteogenic potential compared to traditional lattice designs such as cube and octahedron structures [68]. Similarly, diamond lattices have shown promise in applications requiring high strength-to-weight ratios and controlled deformation [69,70,71]. Recently, negative Poisson’s ratio (NPR) lattices have attracted increasing attention for their bone mimicking properties, which are expected to promote bone tissue regeneration through mechanical stimuli [72]. NPR structures exhibit a unique behavior: they expand transversely when stretched longitudinally and contract transversely when compressed longitudinally. This distinctive property makes NPR materials particularly suitable for applications such as hybrid hip implants. Under bending loads, NPR materials can expand on the side of the implant experiencing tension, addressing challenges faced by traditional hip implants, which tend to shrink on the tension side and expand on the compression side under similar conditions [73]. Moreover, Poisson’s ratio is a critical parameter in biomaterial design, as it plays a vital role in regulating cell behavior, thereby impacting tissue integration and regeneration [74].

In the biomedical field, the design of architected cellular structures is tailored to achieve optimal mechanical compatibility with native tissues, while ensuring biocompatibility and promoting biological functions such as cell attachment and proliferation [75]. LPBF allows for precise customization of lattice geometries, enabling the development of implants and scaffolds that meet the complex demands of personalized medicine.

### 3.2. The Effects of Process Parameters on Dimensional Accuracy

LPBF technology offers significant flexibility in designing lattice structures due to its high design freedom, which is critical for biomedical applications such as bone substitutes and tissue scaffolds. However, achieving dimensional accuracy in the fabrication of lattice structures depends heavily on the careful selection of process parameters. These parameters include laser power, scanning speed, powder characteristics, layer thickness, and scan spacing, which directly affect the accuracy of the final product.

**Figure 9:**
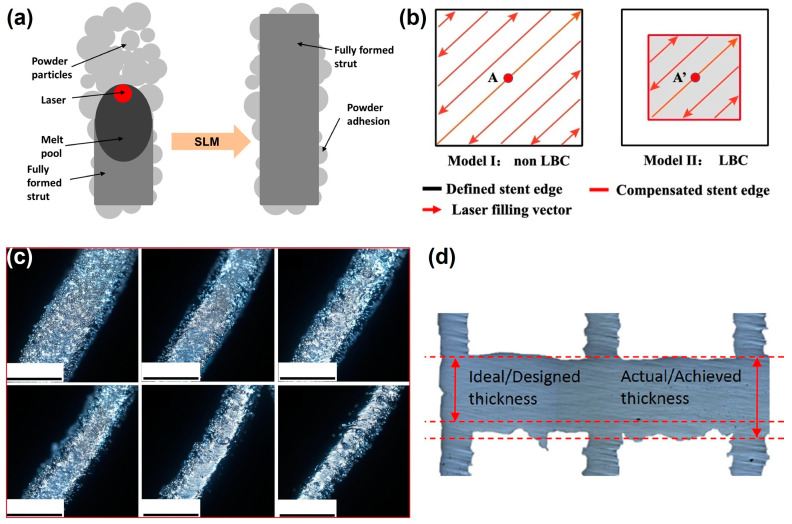
The effects of process parameters on dimensional accuracy: (**a**) forming mechanism of powder adhesions on struts (Adapted with permission from Ref. [77]. Copyright 2018 Elsevier); (**b**) laser filling patterns with and without LBC; (**c**) optical microscope images of NiTi stents at the strut region obtained by using varied LBC values (Adapted with permission from Ref. [78]. Copyright 2023 Springer Nature); and (**d**) actual thickness and design thickness of LPBF Ti6Al4V horizontal strut.

Mazur et al. [76] studied the mechanical properties of Ti6Al4V lattice structures fabricated by LPBF. They found that the mechanical properties of Ti6Al4V differ significantly from theoretical predictions, which suggests that process parameters such as laser power and scanning speed can influence the accuracy of the fabricated structures. Sing et al. [77] further explored the effects of processing parameters on the dimensional accuracy of cellular lattice structures fabricated by LPBF using a titanium–tantalum alloy. They found that the experimental strut dimensions are larger than the designed value, which can be attributed to the lower limit of melt pool size during LPBF (Figure 9a). Moreover, the strut dimensions of the lattice structures are most sensitive to laser power, compared to layer thickness and scanning speed. Li et al. [78] reduced the dimensional deviation of NiTi stent struts between design and as-build specimens to 3% by applying a laser beam compensation (LBC) strategy (Figure 9b,c). However, Gaur et al. [79] claimed that oversizing of horizontal struts cannot be avoided completely, due to the dross accumulation below the horizontal surfaces (Figure 9d).

For cellular structures used in vascular disease and bone tissue engineering, dimensional accuracy is crucial to ensure that the implant fits correctly within the surrounding environment and mimics the mechanical properties of the natural tissue. The precision of cellular structure dimensions, particularly the sizes of the struts and pores, directly impacts the performance of the implant, including its load-bearing capacity and biological performance.

### 3.3. The Effects of Process Parameters on Density

Density is another key factor influencing the performance of lattice structures fabricated by LPBF. The relative density of the structure affects its mechanical properties, such as strength, stiffness, fracture toughness, and fatigue performance, all of which are crucial for biomedical applications, especially in bone replacements and orthopedic implants.

**Figure 10:**
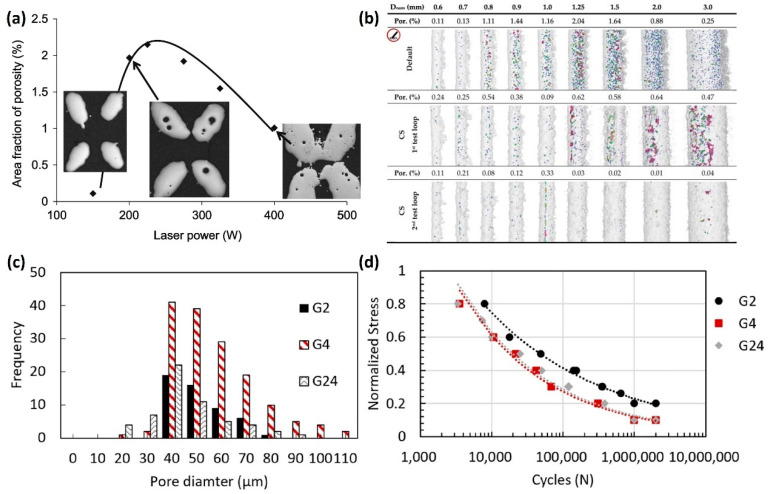
The effects of process parameters on density: (**a**) variation of porosity within the struts as a function of laser power at certain scanning speed for LPBF AlSi10Mg (Apated with permission from Ref. [81]. Copyright 2015 Elsevier); (**b**) comparison of the inclined AlSi10Mg struts produced by the default LPBF process parameters and the contour strategy (Adapted with permission from Ref. [82]. Copyright 2022 Elsevier); (**c**) internal porosity distribution in three Ti6Al4V gyroid lattice structures [83]; and (**d**) S-N curves of the gyroid lattice structures with different internal porosities in (**c**) (Adapted with permission from Ref. [83]. Copyright 2021 Elsevier).

Tsopanos et al. [80] investigated the effects of laser power and exposure time on the relative density of lattice structures. Their results show that relative density increases with the increase in laser power, which leads to more consistent bonding between the powder particles. Higher relative density is beneficial for the mechanical strength of implants, making them more durable and less likely to fail under load. However, excessively high laser power can lead to overheating, resulting in deformation and surface defects. Qiu et al. [81] investigated the effects of laser power and scanning speed on the size and internal porosity of AlSi10Mg lattice struts. Their results reveal a direct correlation between laser power and strut thickness, with increased laser power leading to thicker struts (Figure 10a). Regarding internal density, the highest value was observed at medium laser power and scanning speed. At higher laser power, the melt pools may become turbulent, resulting in pronounced splashing that induces the formation of significant pores and smaller struts, leading to higher porosity. Additionally, when scanning speed is increased, the melt pool becomes more unstable, which is reflected in the development of irregularly shaped struts under these conditions. Thus, the optimized printing parameters for architected cellular structures need to balance the strut density and the dimensional accuracy. Vrána et al. [82] developed contour scanning strategy parameters that led to better elimination of internal pores of lattice structures compared to the traditional hatch scanning strategy (Figure 10b).

Mahmoud et al. [83] investigated the influence of LPBF defects on the fatigue properties of Ti6Al4V gyroids for bone implants. They found that thicker struts have more internal defects than the thinner ones due to higher heat accumulation, and thus less stable melt pools (Figure 10c). As a result, the gyroid scaffolds with thinner struts showed a longer fatigue life than the thicker ones (Figure 10d). For biomedical applications, such as load-bearing implants, optimizing the density and eliminating the internal defects is essential to improve the comprehensive mechanical properties of the scaffolds.

### 3.4. The Effects of Process Parameters on Surface Roughness

Surface roughness plays a critical role in the performance of biomedical implants. The roughness of lattice structures can be influenced by various LPBF process parameters, including laser power, scanning speed, and layer thickness.

**Figure 11:**
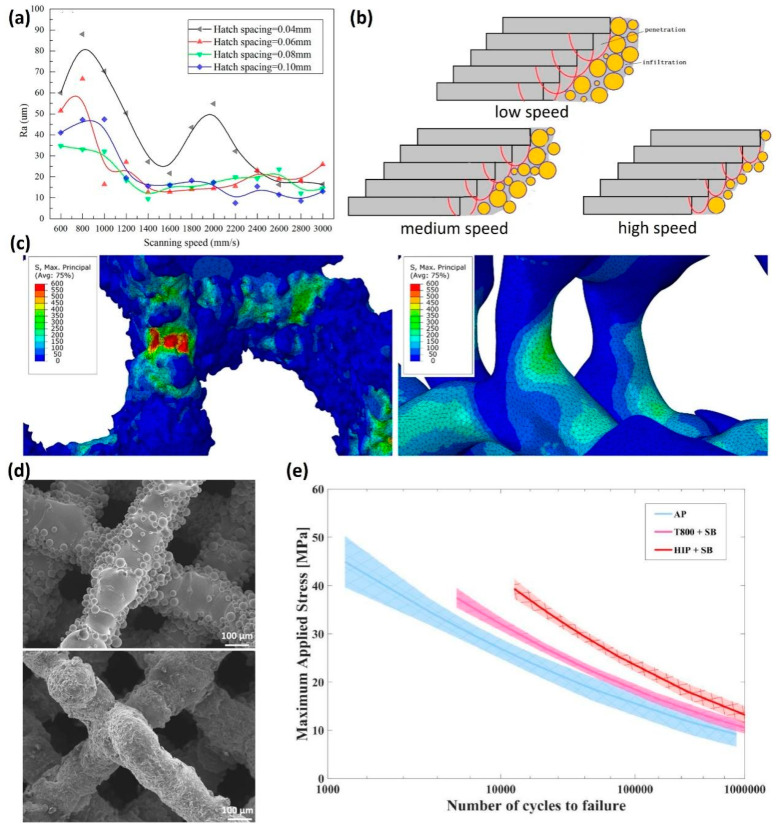
The effects of process parameters on surface roughness: (**a**) arithmetic average roughness (*R_a_*) of lower surface of LPBF AlSi10Mg struts with different processing parameters [85]; (**b**) schematic diagram of lower surface roughness caused by laser penetration and infiltration effect (Adapted with permission from Ref. [85]); (**c**) details of the maximum principal stress distribution for micro-CT model of normal gyroid and CAD model of normal gyroid (Adapted with permission from Ref. [87]. Copyright 2020 Elsevier); (**d**) surface morphology of the LPBF Ti6Al4V samples before and after post treatment [88]; and (**e**) normalized S-N curves of lattice Ti6Al4V structures with different post-treatments (AP: as printed, SB: sand blasted) (Adapted with permission from Ref. [88]. Copyright 2019 Elsevier).

Kadirgama et al. [84] studied the effects of strut shape, size, and porosity on surface roughness. Their findings suggest that surface roughness is primarily influenced by LPBF process parameters rather than the design of the lattice elements themselves. Similarly, Han et al. [85] observed that the strut surface roughness initially decreased and then increased with higher scanning speeds, while the laser power and hatch spacing were kept constant (Figure 11a). This behavior is attributed to the infiltration effect. At lower scanning speeds, the molten pool’s center temperature rises, resulting in a longer lifetime of the molten pool and enhanced wettability and flowability. Consequently, the liquid metal infiltrates the powder gaps more effectively, leading to a smoother surface with reduced roughness (Figure 11b). However, as the scanning speed increases, the infiltration effect weakens, resulting in an uneven surface. With further increases in scanning speed, the infiltration effect diminishes to the point where it is insufficient to bond the powders, ultimately reducing surface roughness again (Figure 11b). Sing et al. [86] also noted that scanning speed impacts powder adhesion on the surface of the struts, which in turn affects surface roughness. While a smoother surface is generally preferred in biomedical applications to reduce wear and improve the comfort of implants, excessive roughness may also be beneficial in certain applications, such as bone scaffolds, where surface roughness can promote cellular attachment and osteointegration. However, for the bone scaffolds used in the load-bearing scenarios where fatigue resistance is important, surface roughness need to be carefully controlled. Yánez et al. [87] studied the effects of surface roughness on the fatigue behavior of LPBF gyroid cellular structures. They found that the topological features of excessive surface roughness of gyroid struts and the surface defects could act as crack initiation sites due to high stress concentration, which is dominant in fatigue damage (Figure 11c). Moreover, Ahmadi et al. [88] investigated different types of post treatment on the fatigue properties of LPBF Ti6Al4V scaffolds. They found that a sandblasting process induced compressive stress on the strut’s surface and removed partially melted particles, leading to substantial increase of the fatigue life of the scaffolds (Figure 11d,e).

**Figure 12:**
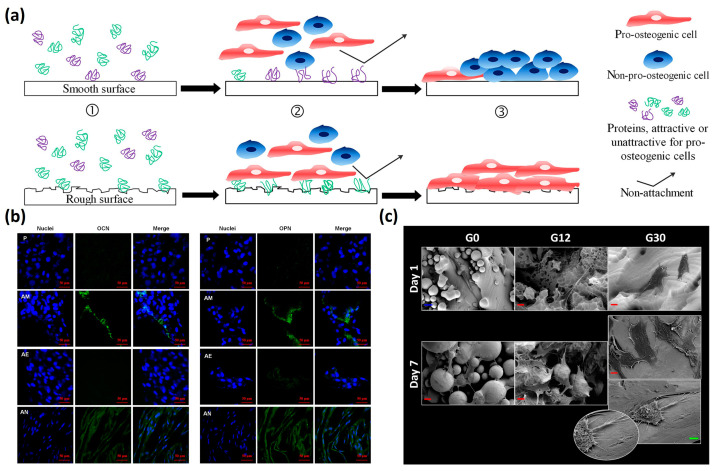
The effect of surface roughness on cell behavior: (**a**) graphic showing the basic cell to material interactions on smooth or textured rough surfaces (Adapted with permission from Ref. [89]); (**b**) immunofluorescent staining of OCN and OPN of MC3T3s after 21 d with different surface treatments on LPBF titanium (P: control, AM: as-built, AE: acid etching, AN: anodization) (Adapted with permission from Ref. [90]); (**c**) MSCs cell morphology is influenced by local topological features (Adapted with permission from Ref. [91]).

For biomedical applications, optimizing surface roughness is essential for both mechanical performance (e.g., improving fatigue properties) and biological integration (e.g., enhancing osteoblast attachment in bone substitutes). Surface roughness is a critical factor influencing the biological interactions between cells, tissues, and biomaterials. Studies have demonstrated that increased hydrophilicity and surface roughness enhance human osteoblast attachment, proliferation, and osseointegration potential compared to hydrophobic and smoother surfaces [89]. Cells interacting with roughened biomaterials exhibit more focal adhesion points, improved cell attachment, and enhanced proliferation (Figure 12a). Ren et al. [90] modified the surface of electron beam melting (EBM) Ti6Al4V implants through acid etching and anodization, successfully removing residual powders and burrs. This process superimposed submicron pits, grooves, and nanotubes onto the original undulating peak/valley surface of the material. The resulting hierarchical micro/nano-structure improved the hydrophilicity and biological activity of the material. Obvious intracellular osteocalcin (OCN) and osteopontin (OPN) expression with a certain directional distribution was detected in the anodization group, indicating that cell differentiation was effectively promoted (Figure 12b). Moreover, local topological features can clearly influence cell morphology. Keeffe et al. [91] explored the effects of chemical etching on the surface of LPBF Ti6Al4V biomaterials with mesenchymal stromal cells (MSCs). They observed that topological nonhomogeneities on the as-built surface, such as partially adhered powder, caused a stretched and anisotropic cell morphology (Figure 12c). As cells spread across the nonhomogeneous powder interface, large unsupported areas formed. Chemical etching gradually removed these surface defects, leading to a more isotropic cell morphology, which is generally conducive to MSC differentiation along an osteoblastic lineage. However, while increased roughness can improve cell adhesion and osseointegration, it can also promote the adhesion of pathogens and biofilm formation. High surface roughness increases the material’s surface area, creating favorable sites for pathogen colonization and biofilm development [92].

### 3.5. The Effects of Process Parameters on Microstructures

**Figure 13:**
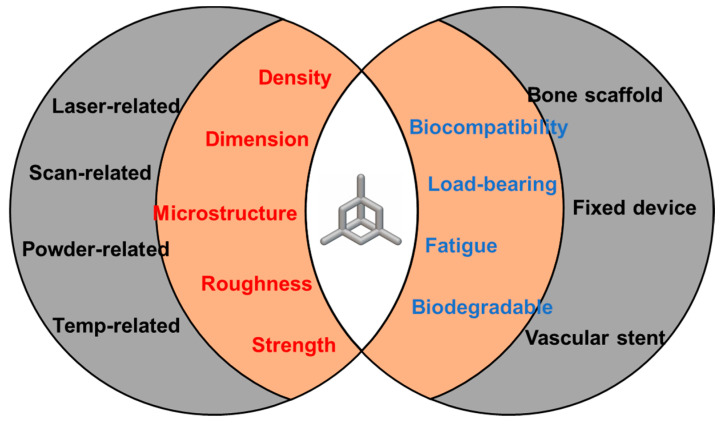
Engineering manufacturing of LPBF scaffolds and their biological relevance.

The microstructure of LPBF-fabricated lattice structures significantly affects their mechanical properties, which are crucial for biomedical applications such as bone implants, where both strength and biocompatibility are required (Figure 13). The microstructure is influenced by process parameters such as laser power, scanning speed, layer thickness, and scanning strategy, which determine the crystallinity, grain size, and phase distribution of the material.

**Figure 14:**
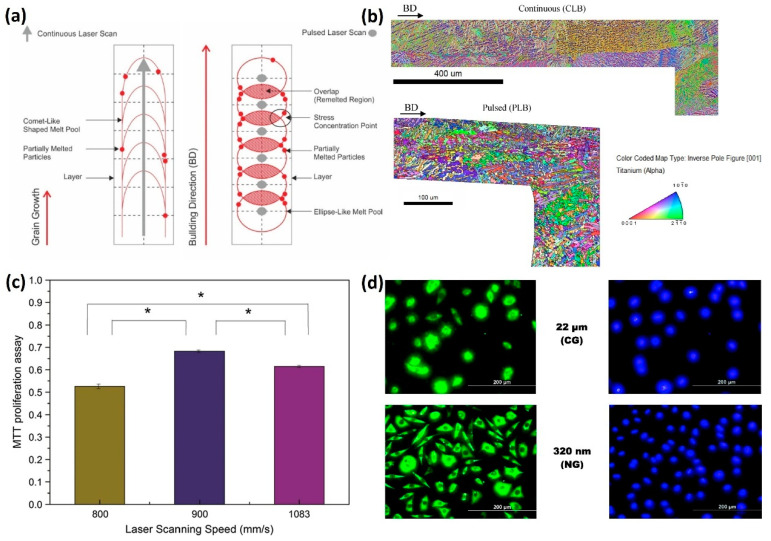
The effects of process parameters on microstructures: (**a**) schematic illustration showing the different laser scanning methods including continuous laser (CL) scan and pulsed laser (PL) scan [93]; (**b**) EBSD inverse pole figure (IPF) maps of LPBF Ti6Al4V specimens with CL and PL scan (Adapted with permission from Ref. [93]); (**c**) MTT proliferation assay of L929 cells grown for 24 h in the extracts of LPBF 316L specimens at three different scanning speeds (Adapted with permission from Ref. [97]. Copyright 2017 Springer nature), (*: Statistically Significant Differences); and (**d**) fluorescence micrographs representing immunocytochemistry of fibronectin (left-hand side) expressed by pre-osteoblasts after incubation for orientations (Adapted with permission 48 h and stained with DAPI (right-hand side) on austenitic stainless steel of different grain sizes (Adapted with permission from Ref. [98] Copyright 2013 Elsevier).

Karami et al. [93] investigated the differences between the continuous and pulsed L-PBF scanning strategies and their effects on the microstructure and mechanical behavior of LPBF Ti6Al4V lattice structures (Figure 14a,b). They found that the size and orientation of acicular α and prior β grains as well as the fraction of retained β were different between continuous and pulsed laser modes, resulting in differences in the hardness, strength, and fatigue life, highlighting the importance of controlling LPBF parameters to design the microstructures. Onal et al. [94] applied single point exposure scanning to fabricate Ti6Al4V scaffolds and found that a re-melting process lead to a finer and more uniform microstructure with higher hardness values. Yang et al. [95] studied the effect of scanning speed on the microstructure of biodegradable Zn. Their findings revealed that, at low scanning speeds, coarse columnar grains dominated with a preferred growth orientation. This phenomenon was attributed to grain epitaxial growth driven by the prolonged high temperature of the molten pool and continuous heat flow. As scanning speed increased, the texture intensity significantly weakened and grain size reduced, indicating that rapid solidification inhibited grain epitaxial growth. The morphology of the microstructure during solidification is determined by the temperature gradient (G) and the solidification rate (R). At low scanning speeds, high heat input strengthened the crystal orientation due to the elevated temperature gradient. Conversely, higher scanning speeds reduced the temperature gradient and increased the solidification rate, resulting in enhanced thermodynamic undercooling. This promoted the nucleation of massive equiaxed dendrites in the supercooled zone ahead of the advancing solid–liquid interface, transforming the preferred oriented grains into randomly oriented grains. However, Xu et al. [96] observed a contrasting phenomenon with LPBF-fabricated Mg alloys. In their study on LPBF NZ30K Mg alloy, they found that grain size decreased with increasing laser energy input. The underlying mechanisms driving this behavior remain unclear and require further investigation. Notably, research on the effects of process parameters on the microstructure of LPBF biodegradable cellular metals is still in its infancy, highlighting the need for more in-depth studies in this area. This is particularly important for biomedical materials, where the microstructure directly influences the material’s strength, fatigue resistance, corrosion behavior, and long-term stability in the body.

In addition to improving mechanical properties, controlling the microstructure of lattice structures also impacts their biocompatibility. Fine-tuning the grain size and phase composition can enhance the material’s interaction with surrounding tissues, promoting better integration and reducing the risk of adverse reactions. For example, Shang et al. [97] found that by changing the scanning speed, the biocompatibility of LPBF 316L specimens can be controlled via microstructure variation (Figure 14c). Misra et al. [98] also observed a much better cellular response to nanograined 316L stainless steel material compared with coarse-grain ones, which can be attributed to the higher hydrophilicity of the substrate and grain structure (Figure 14d). For LPBF biodegradable porous metals, the degradation behavior can also be manipulated through microstructure control with different process parameters [9].

### 3.6. The Interplay Between Structure Design and Process Parameters

**Figure 15:**
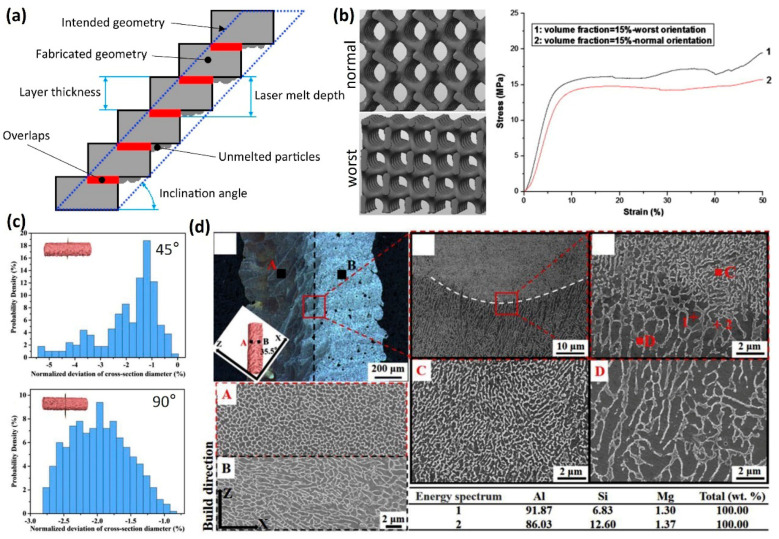
The interplay between structure design and process parameters: (**a**) schematic of inclination angle of the struts (Adapted with permission from Ref. [13]); (**b**) compressive stress–strain curves obtained from the LPBF 316L stainless steel gyroid cellular lattice structures at the normal or worst orientations (Adapted with permission from Ref. [99]. Copyright 2014 Elsevier); (**c**) probability density distributions of LPBF AlSi10Mg normalized deviation for struts with different build orientations (Adapted with permission from Ref. [100]. Copyright 2019 Elsevier); and (**d**) typical optical and SEM (upper zone A and lower zone B of inset) images of the microstructure of the tilted struts and EDX point analyses of the cellular–dendritic network [100].

In addition to printing parameters, the design of lattice structures—including topology, unit cell size, and orientation—plays a pivotal role in determining the quality and mechanical properties of LPBF-fabricated components. During the fabrication of lattice structures using LPBF, limitations often arise, particularly for overhanging surfaces. Without adequate geometric support or deformation control, successful fabrication cannot be achieved. Maconachie et al. [13] demonstrated that the minimum inclination angle required for stable fabrication is influenced by process parameters, material type, and powder characteristics (Figure 15a). Yan et al. [99] explored the feasibility and performance of SS316L gyroid lattice structures, revealing that gyroid configurations with struts oriented at 0 and 90 degrees exhibit better mechanical properties compared to those oriented at 45 degrees (Figure 15b). Furthermore, Dong et al. [100] found that the orientation of the struts has a significant influence on the geometric accuracy and microstructure of LPBF AlSi10Mg cellular structures (Figure 15c,d). This finding underscores the importance of lattice orientation and structural design in optimizing printing parameters and achieving a desirable mechanical performance, particularly for biomedical implants. These insights highlight the necessity of integrating material, process, and design considerations to enhance the functionality and reliability of LPBF-fabricated lattice structures in biomedical applications.

### 3.7. The Integration of Machine Learning on LPBF Process Parameter Optimization

Machine learning (ML) has emerged as a transformative tool for addressing the challenges associated with process parameter optimization in LPBF cellular metallic biomaterials. Its ability to analyze large datasets and uncover complex, nonlinear relationships between parameters and outcomes makes it highly suitable for this purpose.

Xu et al. [101] applied ML to aid real-time detection of keyhole pore generation in the LPBF process. They discovered two types of keyhole oscillation in the laser powder bed fusion of Ti6Al4V, with simultaneous high-speed synchrotron X-ray imaging and thermal imaging, coupled with multiphysics simulations. Amplifying this understanding with ML, they developed an approach for detecting the stochastic keyhole porosity generation events with submillisecond temporal resolution and a near-perfect prediction rate. In addition to the image data, Tempelman et al. [102] utilized a ML model to analyze acoustic signals that were captured during the LPBF process. By extracting features from these signals using advanced decomposition techniques, they employed a support vector machine (SVM) algorithm to classify keyhole porosity with up to 97% accuracy, demonstrating the potential of non-invasive monitoring techniques powered by ML.

**Figure 16:**
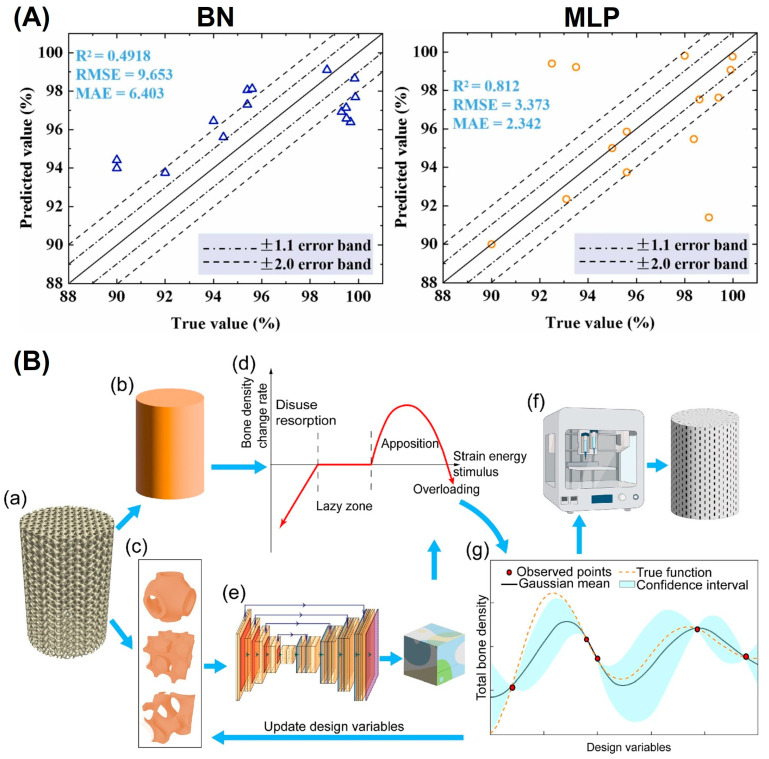
The integration of ML on LPBF cellular metallic biomaterials: (**A**) BN-based and MLP-based model for relative density prediction of LPBF Ti6Al4V alloys (Adapted with permission from Ref. [103]. Copyright 2023 Elsevier); (**B**) schematic of the ML based design framework by coupling with AM to develop subject-specific bone scaffolds: (**a**) An initial bulk scaffold, (**b**) A homogenised bulk scaffold to output macro strain, (**c**) Micro unit cells for optimization, (**d**) Wolff’s law model to evaluate the long-term bone growth results inside the bulk scaffold, (**e**) The U-Net neural network to output micro strain components, (**f**) 3D printing of optimised subject-specific scaffolds using the lithography-based ceramic manufacturing technique, (**g**) Bayesian optimisation (BO) to optimise the structure of micro unit cells. (Adapted with permission from Ref. [105]).

In another study, Shen et al. [103] developed a formability prediction model for Ti6Al4V using Bayesian networks (BN) and multilayer perceptron (MLP) models. The MLP model outperformed BN in predicting the relationship between process parameters and relative density, significantly reducing the time and cost of process optimization (Figure 16A). This validated approach accelerates the engineering application of LPBF-fabricated materials by streamlining the parameter selection process.

Addressing manufacturability challenges, Zhang et al. [104] proposed a novel approach composed of combining a voxel-based convolutional neural network (CNN) and a neural network (NN) to predict potential printing failures. This dual-model framework analyzes both design and process aspects, accurately predicting the manufacturability of LPBF designs under selected process settings, providing a practical tool for enhancing production reliability.

For biomedical applications, Wu et al. [105] developed an ML-based strategy to optimize scaffolds composed of triply periodic minimal surface (TPMS) unit cells (Figure 16B). Using Bayesian optimization, the method enabled time-dependent mechano–biological optimization of scaffolds to meet biomechanical requirements for bone regeneration. Simulation results showed that the optimized scaffolds significantly enhanced bone ingrowth in a segmental defect of a sheep tibia, underscoring the potential of ML to tailor scaffolds for specific clinical needs.

ML has also been employed to predict the mechanical properties of LPBF-fabricated components. Cao et al. [106] constructed a comprehensive database from 173 datasets to optimize process parameters for Ti6Al4V alloys. By integrating clustering techniques with a regression model, they achieved high-precision predictions of material properties. The inclusion of a non-dominated sorting genetic algorithm (NSGA-II) further enabled multi-objective optimization, achieving a superior balance between strength and ductility. Experimental validation confirmed the effectiveness of the optimized parameters, demonstrating the synergy between ML-driven predictions and LPBF experiments.

Additionally, Reddy et al. [107] explored the mechanical performance of LPBF-fabricated lattice structures. Through feature importance analysis and correlation heatmaps, they identified lattice volume and surface roughness as critical predictors of mechanical behavior, accounting for 24.29% and 23.71% of predictive power, respectively. These insights highlight the potential of ML in identifying key factors that influence lattice performance, facilitating the development of tailored designs for specific applications.

In summary, the integration of machine learning into LPBF process parameter optimization is transforming the field by enabling real-time monitoring, efficient prediction of outcomes, and optimization of complex multi-variable systems. These advancements hold great promise for the development of high-performance metallic biomaterials that are tailored to meet the stringent requirements of biomedical applications.

## 4. Summary and Outlook

### 4.1. Summary

LPBF has emerged as a promising additive manufacturing technology for fabricating lattice structures of metallic materials, owing to its unparalleled design freedom. The lattice structures produced by LPBF often exhibit unique properties, such as reduced weight, enhanced energy absorption, and improved mechanical performance, which are not achievable with solid structures. These features make LPBF particularly attractive for biomedical applications, such as bone scaffolds, vascular stents, and prosthetic devices, where customized geometries and lightweight, yet strong, structures are critical.

However, a considerable body of research has shown that variations in the LPBF process parameter can lead to various defects, including dimensional deviation, high surface roughness, and internal porosity. These issues can significantly impact the mechanical properties, such as strength and fatigue performance, which are essential for biomedical implants. Therefore, understanding and optimizing process parameters is critical for ensuring the performance and reliability of LPBF-fabricated lattice structures in medical applications.

This review provides a comprehensive analysis of the interplay between LPBF process parameters, material properties, and architected cellular designs. Key findings underscore the importance of fine-tuning parameters such as laser power, scanning speed, and powder characteristics to achieve the desired dimensional accuracy, porosity, and surface quality. These insights are vital for developing functional and biocompatible implants that meet the rigorous demands of biomedical applications.

### 4.2. Outlook

To address the challenges and unlock the full potential of LPBF for biomedical applications, future research should focus on:

1. Advancing LPBF technology to reduce defects such as porosity and surface roughness remains a priority. Efforts should include refining process parameter optimization to improve mechanical properties, such as fatigue resistance and long-term durability, under physiological conditions.
2. Specific emphasis should be placed on designing LPBF-fabricated lattice structures for critical applications such as load-bearing bone scaffolds, vascular stents, and high-durability prosthetic devices. Addressing application-specific challenges, such as manufacturability, load distribution, biointegration, and degradation control is essential.
3. ML techniques offer transformative potential in optimizing LPBF process parameters. By leveraging large datasets, ML models can predict the influence of parameters on mechanical and biological properties, enabling real-time optimization for various materials and geometries.
4. As demand for customized medical devices grows, ML can support the development of patient-specific lattice structures. By integrating anatomical data, ML could assist in fabricating implants with tailored mechanical properties and porosity, enhancing functionality and longevity.
5. Future research should explore the integration of multi-material and functionally graded designs within LPBF. Multi-material designs can enable the creation of implants with region-specific properties, such as enhanced strength at load-bearing sites and increased porosity for biointegration. Functionally graded structures, where material composition and properties vary spatially, can mimic the hierarchical nature of biological tissues, improving the adaptability and performance of architected cellular structures.
6. Investigating degradable metals, such as magnesium, zinc, and their alloys, is crucial for advancing biomedical applications. These materials offer the advantage of gradual resorption, reducing the need for secondary surgeries. Future studies should focus on understanding and controlling their degradation rates and mechanical properties to ensure safety and efficacy.
7. Future work should also focus on improving the sustainability and cost-effectiveness of LPBF processes, ensuring that high-quality implants are accessible and affordable for a wider range of patients.

The combination of LPBF technology with advanced design methodologies and ML presents an unprecedented opportunity to revolutionize biomedical engineering. This synergy offers a pathway to more efficient, personalized, and cost-effective healthcare solutions, ultimately improving patient outcomes and advancing the frontiers of medical science.

## Figures and Tables

**Table 1:** The impact of different process parameters in LPBF.

Parameter Category	Key Parameters	Effects/Issues	Ref.
Laser-related	Laser power	Density/Keyhole appeared	[32]
	Spot size	Porosity/Cracks appeared	[38]
	Pulse duration	Density/Un-melt	[30]
	Pulse frequency	Density/Pores appeared	[31]
Scan-related	Scanning speed	Density/Un-melt	[39]
	Scan spacing	Density/Balling effect appeared	[40]
	Scan strategy	Density, Residual stress/Micro-structure changed, Un-melt	[43]
Temperature	Bed temp.	Ductility, Solidification changed/Cracks appeared	[50]
	Feeder temp.	Residual stress/Yield strength decreased	[53]
	Temp. uniformity	Grain characteristics/Microhardness changed	[54]
Power-related	Powder size	Manufacturing quality/Surface roughness increased	[57]
	Powder shape and distribution	Porosity/Flowability decreased	[58]
	Material properties	Porosity/Production cost increased	[62]
	Layer thickness	Building efficiency/Surface roughness increased	[61]

## Data Availability

No new data were created or analyzed in this study. Data sharing is not applicable to this article.
